# Advances in α-Lipoic Acid for Disease Prevention: Mechanisms and Therapeutic Insights

**DOI:** 10.3390/molecules30091972

**Published:** 2025-04-29

**Authors:** Yonglian Wang, Shuxia Jiang, Yaoxuan He, Ping Pang, Hongli Shan

**Affiliations:** Shanghai Frontiers Science Research Center for Druggability of Cardiovascular Noncoding RNA, Institute for Frontier Medical Technology, School of Chemistry and Chemical Engineering, Shanghai University of Engineering Science, Shanghai 201620, China; m340122508@sues.edu.cn (Y.W.); hhe@sues.edu.cn (Y.H.); pangping@sues.edu.cn (P.P.)

**Keywords:** α-lipoic acid, biological functions, derivatives, delivery systems, therapeutic strategies

## Abstract

α-Lipoic acid (ALA) is a naturally occurring compound with diverse biological functions, widely distributed in animal and plant tissues. It has attracted considerable attention due to its versatile therapeutic potential. However, despite these promising prospects, the clinical application of ALA remains limited by its low bioavailability and chemical instability and an incomplete understanding of its multifaceted mechanisms across various diseases. This review provides a comprehensive overview of the biochemical properties of ALA, including its direct free-radical-scavenging activity, regeneration of endogenous antioxidants, chelation of metal ions, and modulation of inflammatory responses. We also highlight the current evidence regarding ALA’s therapeutic roles and efficacy in major diseases, such as neurodegenerative disorders, lung diseases, cardiovascular diseases, and diabetes. Furthermore, recent advancements and innovative strategies in ALA-based derivatives and drug-delivery systems are summarized, emphasizing their potential to address complex diseases and the necessity for further translational studies. This review aims to provide a theoretical foundation for the rational design of ALA-based therapies, thereby supporting future clinical applications and the optimization of therapeutic strategies.

## 1. Introduction

**Figure 1:**
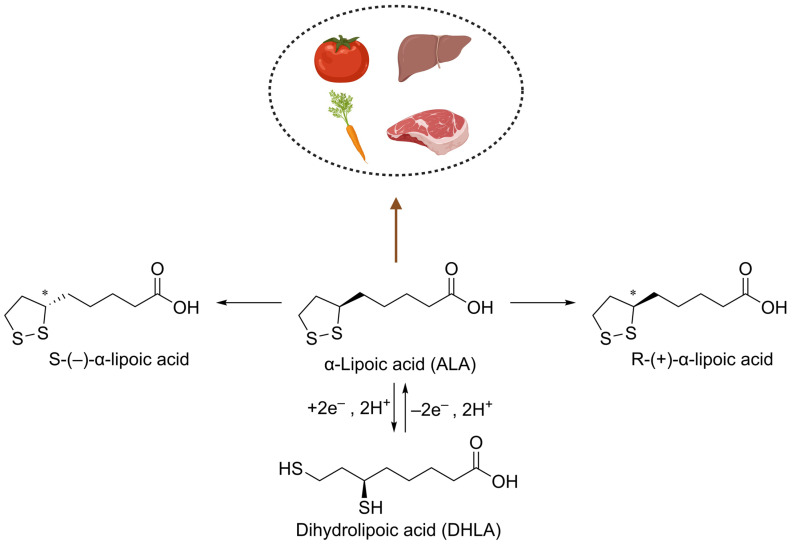
The source and chemical structure of ALA. ALA is an antioxidant found in foods like tomatoes, carrots, and meat. It is converted to DHLA through redox reactions. The chiral centers in ALA results in two enantiomers: S-(−)-α-lipoic acid and R-(+)-α-lipoic acid. * Carbon chiral center.

α-Lipoic acid (ALA), also known as lipoic acid (LA), is a naturally occurring bioactive compound with multifunctional therapeutic properties, first isolated from the liver tissue in 1951 by Reed et al. [1]. The single chiral center at the C6 position of ALA results in two enantiomeric forms: (S)-ALA and (R)-ALA. The R-ALA is a naturally occurring form and predominates in mediating the biological activity of ALA [2]. ALA and its reduced form, dihydrolipoic acid (DHLA), form a stable redox pair, which is interconverted in vivo through enzymatic pathways, including disulfide dehydrogenase, thioredoxin reductase, and glutathione reductase [3]. ALA is found in various food sources, including red meat and plants [4]. Due to its hydrophilic and hydrophobic properties, ALA can function effectively in cell membranes and the cytoplasm [5]. Although dietary sources of ALA are limited, exogenous intake can enhance its systemic levels, thereby supporting its therapeutic applications [6] (Figure 1).

This review presents a comprehensive analysis of the diverse therapeutic potential of ALA by integrating the recent advancements. It provides an updated perspective on ALA-related therapies, with a particular emphasis on its applications in the treatment of neurodegenerative disorders, pulmonary diseases, cardiovascular conditions, and diabetes. In addition, the review highlights the development of novel therapeutic strategies, including ALA derivatives and advanced drug delivery systems, underscoring their promise in clinical applications. Furthermore, it emphasizes the necessity of the further optimization of delivery systems and translational applications. By bridging mechanistic insights with therapeutic innovation, this review aims to offer new directions for ALA-based interventions and support advances in effective clinical treatment strategies.

## 2. Biological Functions of ALA

**Figure 2:**
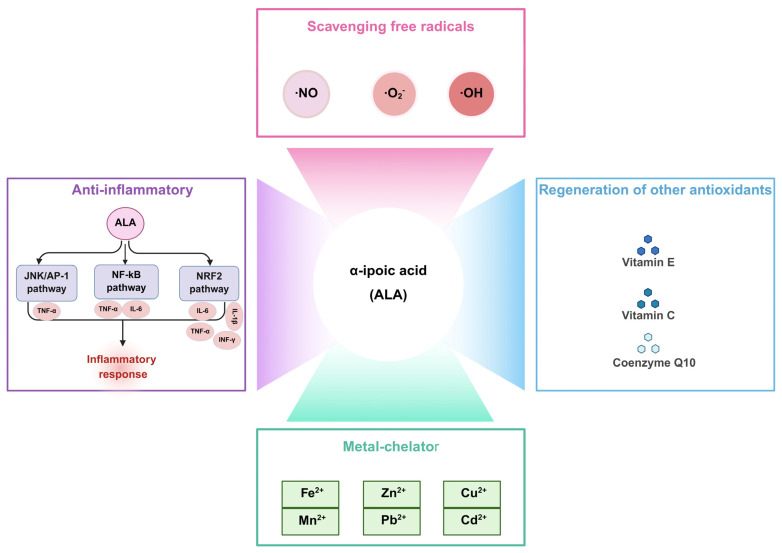
Multiple biological functions of ALA. ALA exerts antioxidant effects by scavenging reactive species, including •NO, •O_2_^−^, and •OH. It also inhibits inflammation by modulating the JNK/AP-1, NF-κB, and NRF2 pathways, which reduce the expression of inflammatory factors, such as TNF-α, IL-6, IL-1β, and INF-γ. Additionally, ALA can regenerate other antioxidants, including vitamin C, vitamin E, and coenzyme Q10. As a metal chelator, ALA binds to metal ions (Fe^2+^, Zn^2+^, Cu^2+^, Mn^2+^, Pb^2+^, and Cd^2+^).

ALA is the only known antioxidant with both water and lipid solubility, known as the “universal antioxidant”. This unique property enables ALA to enhance the body’s antioxidant capacity through various mechanisms, including the direct scavenging of free radicals, regeneration of endogenous antioxidants, chelation of metal ions, and modulation of inflammatory responses (Figure 2).

### 2.1. Scavenging Free Radicals

Previous studies discovered that reactive oxygen species (ROS), reactive nitrogen species (RNS), and highly reactive free radicals (•OH, •O_2_^−^, •NO) produced during aerobic metabolism or stress responses can be effectively neutralized by ALA through direct hydrogen donation or redox cycling mechanisms [7]. It has been reported that the addition of ALA in sperm washing media maintained sperm viability and motility by reducing ROS generation and preserving sperm DNA integrity [8]. Furthermore, supplementation with two antioxidants, ALA and N-acetyl-l-cysteine, has been shown to reduce retinal ROS levels and repair damaged mitochondria, thereby improving retinal functions. These findings support the potential of antioxidant supplementation as an effective intervention strategy for individuals experiencing sleep deprivation [9]. In an Alzheimer’s disease model, ALA has been demonstrated to inhibit the formation of •OH and reduce the expression of inducible nitric oxide synthase (iNOS) and nitric oxide (NO), thereby indirectly mitigating disease progression [10]. Additionally, DHLA effectively scavenges superoxide anions and peroxyl free radicals, preventing free radical-mediated protein oxidation [11]. Therefore, ALA is considered to exert cytoprotective effects against oxidative stress by scavenging these reactive species.

### 2.2. Regeneration of Other Antioxidants

Antioxidants are oxidized during the process of neutralizing free radicals, temporarily losing their scavenging ability until they are subsequently reduced. DHLA, as the reduced form of ALA, mitigates oxidative damage by regenerating endogenous antioxidants, including vitamin E, vitamin C, and glutathione (GSH) [12]. Moreover, the DHLA-mediated reduction of the oxidized form of coenzyme Q10 contributes to the reduction of α-tocopherol free radical generation [13]. Therefore, ALA provides valuable therapeutic approaches for diseases related to free radicals by increasing cellular glutathione levels and participating in the redox of antioxidants (vitamins E and C) [5].

### 2.3. Metal Chelator

The ability of ALA to form complexes with redox-active metal ions has been well established. Previous studies have demonstrated that ALA effectively chelates divalent metal ions, including Cu^2+^, Fe^2+^, Zn^2+^, Mn^2+^, Cd^2+^, and Pb^2+^, which in turn contributes to the prevention and treatment of cellular toxicity caused by metal ion deposition [14]. ALA treatment has been shown to reverse iron deposition, oxidative stress, and the increase in autophagy induced by ferrous ammonium citrate [15]. In the context of copper-induced neurotoxicity, the co-administration of monoisopropyl-DMSA (MiADMSA) and ALA provides a more effective treatment strategy than either MiADMSA or ALA alone [16]. In summary, ALA offers therapeutic benefits by reducing metal-induced oxidative damage and enhancing antioxidant activity through metal chelation.

### 2.4. Anti-Inflammatory

Inflammation is a consequence of an innate biological response triggered by an organism’s exposure to harmful stimuli. It is a protective mechanism to eliminate injurious factors and preserve tissue integrity. However, when inflammation becomes chronic, it contributes to the development and progression of various diseases [17]. ALA has been demonstrated to exert anti-inflammatory effects by directly modulating specific signaling pathways. It is reported that ALA protects against hepatic inflammation, as evidenced by reduced hepatic levels of tumor necrosis factor-α (TNF-α) and interleukin-6 (IL-6), which are associated with the upregulation of nuclear factor erythroid 2-related factor 2 (NRF2) and inhibition of nuclear factor-κB (NF-κB) signaling [18,19]. In addition, treatment with dihydrolipoic acid-coated gold nanoclusters (DHLA-Au NCs) inhibits the activation of JNK and its downstream target c-Jun, subsequently suppressing AP-1-mediated TNF-α expression, thereby contributing to the attenuation of cellular inflammation and senescence [20]. Costa et al. [21] found that ALA alleviates irinotecan-induced inflammation in the duodenum, characterized by elevated levels of IL-6 and IL-1β.

## 3. Pharmacological Application of ALA

The antioxidant, anti-inflammatory, and metal-chelating properties of ALA have positioned it as a key candidate for the therapy of various diseases, including neurodegenerative disorders, pulmonary fibrosis, lung cancer, and cardiovascular diseases, providing a theoretical basis for clinical applications. Although clinical studies remain limited, existing data and experimental findings have demonstrated its potential therapeutic benefits. Additionally, ALA has been shown to effectively improve diabetic symptoms, alleviate diabetic neuropathy, and prevent the development of diabetic cataracts.

### 3.1. Neurodegeneration

Neurodegenerative diseases (NDs) are a group of neurological disorders characterized by the loss of neuronal function in the central or peripheral nervous system, leading to functional impairment [22]. Due to the heterogeneity of clinical manifestations and the diversity of potential pathogenic mechanisms, there is an urgent need for effective interventions to overcome the challenges in developing therapeutic strategies for NDs [23]. Recent studies have reported promising findings about the potential therapeutic effects of ALA in NDs, which are summarized in Table 1.

#### 3.1.1. Alzheimer’s Disease

**Figure 3:**
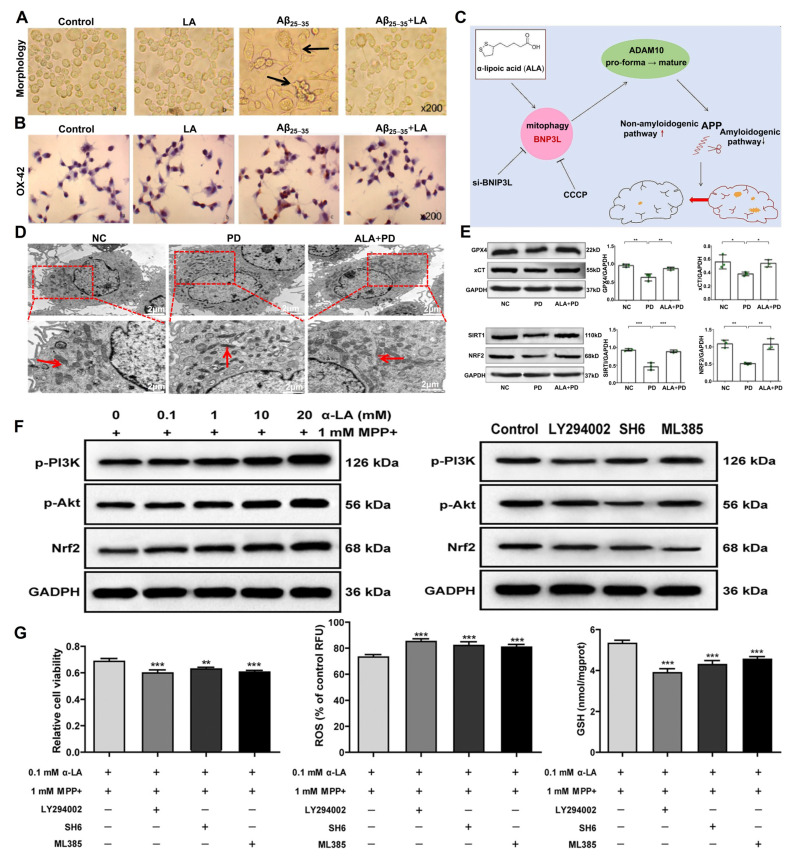
The role of ALA in neurological diseases. (**A**) ALA alleviated the morphological changes in BV2 cells induced by Aβ_25–35_, including cell aggregation, volume increase, spindle shape, and cellular protrusions. Black arrows indicate the representative morphological changes. (**B**) ALA inhibited the activation of BV2 cells caused by Aβ_25–35_ [27]. Copyright: Mol. (Basel Switz.) 2023. (**C**) ALA mediated mitochondrial autophagy through the activation of BNIP3L, increasing the expression of ADAM10 and α-secretase activity, thereby ameliorating the cognitive impairment in APP23/PS45 transgenic mice [29]. Copyright: Alzheimer’s Res. Ther. 2024. (**D**) ALA rescued the mitochondrial damage associated with ferroptosis in PD. Red arrows indicate the chondriosome. Scale bars: 2 μm. (**E**) ALA improved the protein expression of ferroptosis-related proteins and SIRT1/NRF2 signaling pathway in PD. * *p* < 0.05, ** *p* < 0.01, *** *p* < 0.001. Reproduced with permission [30]. Copyright: Neurosci. Lett. 2023. (**F**,**G**) ALA ameliorated MPP+-induced ferroptosis in PC12 cells by regulating the PI3K/AKT/NRF2 pathway. LY294002, PI3K inhibitor; SH6, AKT inhibitor; ML385, NRF2 inhibitor. ** *p* < 0.01; *** *p* < 0.001. Reproduced with permission [35]. Copyright: Cell Biol. Int. 2021. BNIP3L, BCL-2/E1B19kDa-interactingprotein3-like; ADAM10, A disintegrin and metalloproteinase 10; SIRT1, silence information regulator 1; NRF2, nuclear factor (erythroid-derived 2)-like 2; PI3K, phosphatidylinositol 3-kinase; AKT, RAC-alpha serine/threonine-protein kinase.

Relatively rare but noteworthy reports have found that daily administration of ALA (600 mg) to 43 patients with Alzheimer’s disease (AD) significantly slowed disease progression in those with mild dementia over a 48-month observation period [36]. Furthermore, another clinical study reported that treatment with 600 mg/day ALA combined with omega-3 fatty acids significantly attenuated cognitive and functional decline in 39 AD patients over 12 months [37]. In recent years, evidence suggested that ALA improves mitochondrial dysfunction in AD cells by increasing the activity of diversified complexes in the respiratory chain, thereby enhancing mitochondrial membrane potential (MMP) and increasing ATP levels [24]. Metsla et al. [25] reported that ALA supplementation facilitates the transport of copper from extracellular to intracellular, alleviating the intracellular copper deficiency of AD neurons. Moreover, ALA has been shown to protect against the development of AD by improving the motor activity of fruit flies with human Aβ overexpression. Another study demonstrated that ALA administration significantly ameliorates scopolamine-induced memory impairment and exploratory behavior by reducing acetylcholinesterase activity and correcting the abnormal levels of amines in the hippocampus and prefrontal cortex [26]. Additionally, ALA exhibited neuroprotective effects by effectively inhibiting apoptosis, morphological changes, and inflammatory responses in BV2 (Figure 3A,B) [27], as well as regulating the Wnt/β-catenin pathway in PC12 cells activated by Aβ_25–35_ [28]. Mechanistically, ALA improved the cognitive deficits of APP23/PS45 transgenic mice by promoting the maturation of ADAM10 and α-cleavage of APP via BNIP3L-mediated autophagy (Figure 3C) [29]. Therefore, targeting anti-inflammation and mitochondrial functions may represent a promising strategy for the treatment of AD.

#### 3.1.2. Parkinson’s Disease

Previous studies have shown that ALA alleviates the motor deficits in Parkinson’s disease (PD) models by regulating iron metabolism, including the upregulation of ferritin heavy chain (FTH1) and ferroportin (FPN), as well as the downregulation of divalent metal transporter 1 (DMT1). Additionally, ALA mitigates ferroptosis through the SIRT1/NRF2 signaling pathway (Figure 3D,E) [30]. Notably, ALA has been shown to promote the survival of dopaminergic neurons and mitigate motor deficits by decreasing the intracellular ROS and iron levels, as well as inhibiting the decline in superoxide dismutase (SOD) activity and tyrosine hydroxylase (TH) expression [31]. Exposure to ALA, an activator of PGC1α, enhanced the cognitive function in PD rats by increasing mitochondrial biogenesis and modulating neuroinflammatory pathways, accompanied by reduced oxidative stress and inflammatory levels [32]. Moreover, ALA prevented 6-tetrahydropyridine-induced neurotoxicity in vitro and in vivo by upregulating the expression of SIRT1 and PGC1α [33]. Additionally, MPTP-induced motor dysfunction in mice was significantly alleviated by ALA through a reduction in NF-κB, TNF-α, and iNOS in the substantia nigra and spinal cord [34]. In PC12 cells, ALA alleviated the decrease in cell viability and ferroptosis induced by MPP+ through stimulation of the PI3K/AKT/NRF2 pathway (Figure 3F,G) [35].

This evidence strongly supports the therapeutic potential of ALA in NDs, highlighting its ability to enhance mitochondrial function, regulate iron metabolism, modulate inflammation, and prevent apoptosis. These properties, coupled with its capacity to interact with critical signaling pathways, position ALA as an innovative therapeutic strategy for complex NDs. Future clinical trials are essential to determine how these preclinical benefits translate into the treatment of human diseases, as well as exploring the potential of combination therapies for optimizing therapeutic efficacy.

### 3.2. Pulmonary Diseases

Lung cancer is the leading cause of cancer-related death, with its incidence and mortality rates rising annually, posing a substantial threat to human life [38]. Pulmonary fibrosis (PF) is a chronic, interstitial lung disease characterized by fibroblast proliferation and excessive extracellular matrix (ECM) deposition, leading to a gradual decline in lung function and respiratory distress. Notably, the relative incidence of lung cancer is appreciably higher in patients with PF compared to those without PF, with reported rates ranging from 4.4% to 13% and as high as 48% in autopsy samples [39]. Therefore, early intervention and integrated management strategies that address PF and lung cancer could improve patient survival and quality of life. In recent years, accumulating preclinical studies have investigated the role of ALA in both pulmonary fibrosis and lung cancer (Table 2).

#### 3.2.1. Pulmonary Fibrosis

**Figure 4:**
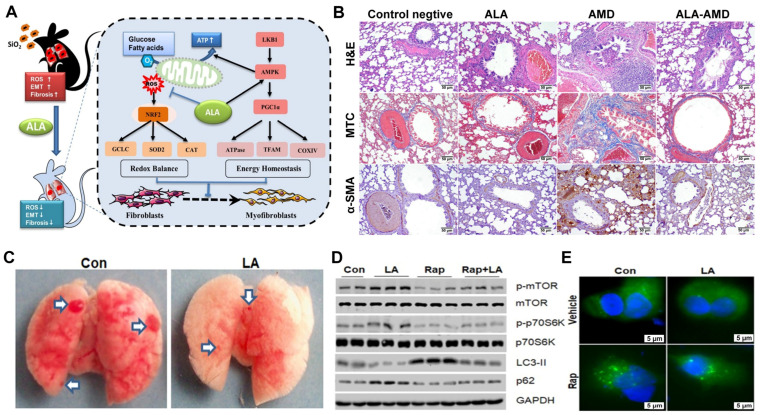
The role of ALA in pulmonary diseases. (**A**) ALA attenuated lung fibrosis by regulating the NRF2 and AMPK/PGC1α pathways, promoting expression of the antioxidant enzyme CAT and energy metabolism-related proteins and inhibiting fibroblast-to-myofibroblast transformation. Reproduced with permission [40]. Copyright: Toxicol. Lett. 2021. (**B**) The ameliorative effect of ALA on AMD-induced histological changes in lung tissue, including pneumonia, collagen deposition, and the expression of α-SMA. Scale bars: 50 μm. Reproduced with permission [42]. Copyright: Mol. Cell. Biochem. 2021. (**C**) ALA suppressed the growth of lung cancer. Arrows indicate the tumor nodules. (**D**,**E**) Inhibition of mTOR reversed the ALA-induced activation of mTOR and inhibition of autophagy in A549 lung cancer cells. Scale bars: 5 μm [46]. Copyright: FEBS Open Bio 2020. ROS, reactive oxygen species; EMT, epithelial–mesenchymal transition; GCLC, glutamate cysteine ligase catalytic; SOD, superoxide dismutase; CAT, catalase; LKB1, liver kinase B1; AMPK, adenosine monophosphate-activated protein kinase; PGC1α, peroxisome proliferator-activated receptor γ coactivator 1α; ATPase, adenosine triphosphate synthase; TFAM, mitochondrial transcription factor A; COX, cytochrome c oxidase; AMD, amiodarone; α-SMA, α-smooth muscle actin; mTOR, mammalian target of rapamycin. Rap, rapamycin.

Exogenous ALA was found to alleviate silica-induced pulmonary fibrosis through activation of the AMPK/PGC1α pathway, which enhanced mitochondrial function and reduced the production of ROS in mice (Figure 4A) [40]. Yan et al. [41] reported that the low expression of lipoic acid synthase (LIAS) aggravated pulmonary fibrosis caused by SiO_2_, while ALA supplementation promoted an immune balance between Th17 and Treg responses, reducing the accumulation of ECM and inflammatory cells in lung tissues, alleviating silicosis fibrosis. Furthermore, treatment with ALA and the endogenous overexpression of LIAS mitigated chronic inflammatory responses and enhanced antioxidant defenses, protecting against amiodarone- (Figure 4B) [42] or silica-induced pulmonary fibrosis [43]. Elhadidy et al. [44] discovered that ALA alleviated the pulmonary cytotoxicity of busulfan through the upregulation of cyclooxygenase-2 (COX-2) and downregulation of NADPH oxidase-4 (NOX-4) expression. The lung injury following radiation was mitigated by ALA, as indicated by the reduced infiltration of most inflammatory cells, edema, alveolar damage, and fibrosis [45]. This highlights the potential of ALA as a versatile player by simultaneously addressing the inflammation and fibrotic components in pulmonary fibrosis.

#### 3.2.2. Lung Cancer

As an antioxidant, ALA has attracted considerable attention in cancer research. It has been shown that ALA inhibits the progression of lung cancer by suppressing autophagy in A549 cells, a process mediated by activation of the mTOR/p70S6K pathway (Figure 4C–E) [46]. Moreover, ALA depletes Oct-4 and β-catenin by reducing the levels of p-AKT, thereby inhibiting epithelial–mesenchymal transition (EMT) in non-small cell lung cancer (NSCLC) cells [47]. Yang et al. [48] demonstrated that ALA inhibits tumorigenesis by reducing GRB2-mediated EGFR and ERK1/2 phosphorylation in NSCLC. Additionally, ALA sensitized NSCLC cells to apoptosis by downregulating the expression of PDK1, leading to a reduction in NRF2 levels and an enrichment of mitochondrial ROS [49]. In combination with cisplatin, etoposide, and paclitaxel, ALA increased the sensitivity of lung cancer cells to apoptosis by downregulating integrin β1 and β3, which are related to invasive behavior and metastasis [50].

Taken together, the present findings emphasize the versatility of ALA in the therapy of pulmonary fibrosis and lung cancer. Its dual ability to modulate the redox balance and immune responses highlights the therapeutic promise of ALA in addressing these complex diseases. However, these studies assume a linear pathway of action, but the complexity of ROS-mediated pathways and the potential for off-target effects in humans remains to be fully elucidated.

### 3.3. Cardiovascular Diseases

Cardiovascular diseases represent a serious threat to global health, characterized by high incidence, substantial disability, and elevated mortality, making it the leading cause of death worldwide [51]. Recent studies have highlighted the potential of ALA as a cardioprotective agent, as summarized in Table 3.

#### 3.3.1. Myocardial Infarction

**Figure 5:**
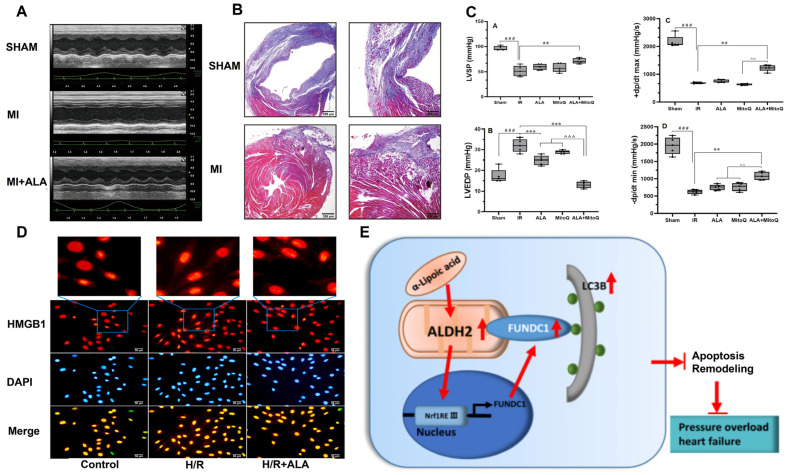
The role of ALA in cardiovascular diseases. (**A**,**B**) ALA improved cardiac function (cchocardiography) and myocardial fibrosis (Masson staining). Scale bars: 250 μm. Reproduced with permission [53]. Copyright: Int. Immunopharmacol. 2023. (**C**) ALA in combination with Mito Q improved cardiac function in IR mice, including the LVSP, LVEDP, LV dp/dt max, and LV dp/dt min. ** *p* < 0.01, *** *p* < 0.001 vs IR group. ### *p* < 0.001 vs sham group. ^^ *p* < 0.01, ^^^ *p* < 0.001 vs Mito Q and ALA groups. Reproduced with permission [54]. Copyright: Exp. Gerontol. 2024. (**D**) ALA alleviated H/R-induced high expression of HMGB1 in H9c2 cells. Reproduced with permission. Scale bars: 50 μm. [60]. Copyright: Eur. J. Pharmacol. 2022. (**E**) ALA promoted autophagy to ameliorate pressure-overload-induced heart failure by activating FUNDC1 in an ALDH2-dependent manner [62]. Copyright: Cell Death Dis. 2020. IR, ischemia–reperfusion; LVSP, left ventricular systolic pressure; LVEDP, left ventricular end-diastolic pressure; LV, left ventricular; H/R, hypoxia/reoxygenation; HMGB1, high mobility group box 1; FUNDC1, FUN14 domain containing 1; ALDH2, acetaldehyde dehydrogenase 2.

The daily oral administration of ALA has been shown to significantly reduce oxidative stress and improve the left ventricular ejection fraction (LVEF) and left ventricular end-systolic volume (LVESV) in mice with acute myocardial infarction (AMI). Moreover, the survival rate of mice with AMI treated with ALA was increased by 63% [52]. Furthermore, ALA attenuated MI by inducing M2b macrophage polarization through HMGB1/NF-κB-pathway-mediated inflammation, oxidative stress, autophagy, and apoptosis, providing a hopeful strategy for the treatment of MI (Figure 5A,B) [53]. Nemati et al. [54] proposed that the combined intervention of Mito Q and ALA synergistically improves cardiac function in aged MI rats by suppressing inflammation and apoptosis (Figure 5C). Additionally, supplementation with ALA was found to improve isoproterenol-induced MI injury, reducing mortality and the incidence of ventricular arrhythmias in experimental MI [55].

#### 3.3.2. Myocardial Ischemia–Reperfusion

A recent study has highlighted the significant therapeutic potential of combining MitoQ and ALA in protecting aged rats from ischemia/reperfusion (IR) injury by enhancing oxidative stress resistance and improving mitochondrial functions in aged rats [56]. In diabetic hearts, ALA pretreatment significantly restored postconditioning-induced cardiac protection by modulating oxidative stress, autophagy, and the recovery of mitochondrial function [57,58]. In addition, the simultaneous use of ALA and postconditioning promoted the recovery of diabetic hearts after I/R by inhibiting apoptosis, as evidenced by increased protein expression of cleaved Caspase-3 and BAX, alongside reduced expression of BCL-2 [59]. Moreover, Qi et al. [60] reported that ALA regulated the translocation of HMGB1 through the HMGB1/TLR4/NF-κB pathway, preventing apoptosis and oxidation, thus inhibiting myocardial IR injury (Figure 5D). Therefore, the combination of ALA with other interventions may serve as an effective strategy to attenuate cardiac I/R damage.

#### 3.3.3. Heart Failure

Cardiac remodeling, driven by various cardiovascular diseases, can progress to heart failure in advanced stages. Although limited, some studies have explored the therapeutic effects of ALA on heart failure. Pop et al. [61] demonstrated that intermittent ALA treatment alleviates glucose and lipid metabolic disorders in rats, protecting against heart failure through known antioxidant effects and its inherent anti-obesity and anti-inflammatory properties. Furthermore, ALA was shown to mitigate left ventricular hypertrophy and dysfunction induced by transverse aortic constriction (TAC) in mice. This protective effect was attributed to the activation of the NRF1/FUNDC1 pathway, which enhanced the activity and expression of ALDH2 (Figure 5E) [62].

In summary, current findings highlight the multifaceted cardioprotective properties of α-lipoic acid in mitigating myocardial infarction, ischemia-reperfusion injury, and heart failure. ALA’s actions extend beyond mere antioxidant effects, influencing key signaling pathways involved in inflammation, apoptosis, and metabolic regulation. The combination of ALA with other therapeutic agents, such as MitoQ, further enhances its cardioprotective potential. However, further comprehensive and rigorous studies are required to confirm these effects and fully elucidate the underlying mechanisms.

### 3.4. Diabetes

Metabolic diseases, particularly diabetes and its associated complications, profoundly impair individuals’ quality of life. Among these complications, diabetic polyneuropathy (DPN) and diabetic retinopathy are especially prevalent, leading to the significant deterioration of neurological functions and visual acuity. Accumulating studies have elucidated the underlying mechanisms and clinical applications of ALA in attenuating the progression of diabetes and its complications (Table 4), highlighting its potential as a promising therapeutic agent in the management of diabetes.

DPN is closely associated with elevated cellular ROS production and the extent of endothelial dysfunction in patients with T2DM [63,64]. ALA has been demonstrated to alleviate DPN symptoms by reducing oxidative stress and improving microcirculatory functions. The Neurological Assessment of Thioctic Acid in Diabetic Neuropathy (NATHAN 1) trial showed that the long-term oral administration of ALA over a period of 4 years produced clinically meaningful improvements and modestly delayed the progression of neurological deficits in patients with DPN [65]. Additionally, a 6-month intervention with oral ALA (600 mg/day) significantly reduced advanced glycation end products (AGEs), increased the current perception threshold and granulocyte protein levels, and decreased asymmetric dimethylarginine (ADMA), thereby improving vascular endothelial function and alleviating the neuropathic symptoms of type 2 diabetes mellitus (T2DM) patients with neuropathy [66]. In a randomized, single-center, double-blind, placebo-controlled clinical trial, the oral administration of 600 mg/kg ALA twice daily for over 6 months resulted in significant symptomatic improvements in DPN patients. Moreover, mild nausea was reported in six patients. No participants discontinued treatment. This clinical trial further confirmed the efficacy, safety, and tolerability of ALA for the treatment of DPN [67]. In a study involving 1242 patients, the administration of different doses of ALA (600, 200, and 1800 mg/day) significantly alleviated sensory symptoms and favorably impacted diabetic sensorimotor peripheral neuropathy (DSPN). A dose-dependent improvement was observed in both the total symptom score (TSS) and the global satisfaction score compared with the placebo [68]. Furthermore, in a study of 54 T2DM patients with DSPN, six months of ALA administration (600 mg/day) resulted in decreased levels of serum kallistatin, TNF-α, and ADMA. A positive correlation was identified between changes in kallistatin and oxidized low-density lipoprotein (oxLDL) levels. These findings suggest that kallistatin may serve as a potential biomarker for evaluating the therapeutic response to ALA in patients with DSPN [69].

Individuals with diabetes have a 25-fold increased risk of blindness and early cataracts compared to the general population, with approximately 20% of diabetic patients developing cataracts [70]. Although relatively uncommon, several reports have highlighted the potential of ALA to delay the formation of diabetic cataracts. It is reported that the weekly administration of ALA at a dose of 10 mg/kg in diabetic mice reduced the incidence of grade 2 cataract formation from 100% to 71% [71]. The primary mechanism underlying cataract development in diabetes involves the generation of sorbitol, a sugar alcohol produced via the action of aldose reductase (AR). The inhibition of AR is therefore considered a promising strategy for preventing diabetic cataracts. ALA, acting as both an antioxidant and an AR inhibitor, has been shown to delay the development and progression of diabetic cataracts by reducing oxidative stress and potentially inhibiting protein glycation [72]. A prospective, placebo-controlled, double-blind study demonstrated that oral administration of Ocu-GLO Rx™, a commercially available antioxidant formulation containing ALA and other antioxidants, exerted beneficial effects on the reduction in cataract progression in diabetic dogs [73]. Notably, diabetic dogs supplemented with ALA alone exhibited a decrease in lens opacities and a significant delay in cataract formation, thereby slowing and potentially preventing the onset of cataract [74]. These findings suggest that by inhibiting AR activity and modulating the polyol pathway, ALA may play an important role in the prevention and treatment of diabetic cataracts.

**Table 4:** Therapeutic effects of ALA in diabetes and its complications.

Research Object	ALA Treatment	Outcomes	Reference
460 patients with mild/moderate DSPN	600 mg/kg/day, orally, 4 years	↑NIS, NIS-LL, NSC	[65]
54 T2DM patients	600 mg/kg/day, orally, 6 months	↑NO; ↓AGEs, ADMA, TNF-α, CAS, DN4, CPT	[66]
200 patients with DPN	600 mg/kg, twice-daily, orally, 6 months	↑NSS, NDS, VAS, VPT	[67]
1242 patients with DSPN	600/800/1200 mg/kg/day, orally, 24 months	↑TSS, NDS, NIS, and the global satisfaction score	[68]
54 T2DM patients with DSPN	600 mg/kg/day, orally, 6 months	↑NO; ↓serum kallistatin, TNF-α, ADMA, NTSS-6, DN4	[69]
28 male BALB/C mice	10 mg/kg, intraperitoneally weekly, 5 weeks	↓grade 2 cataract	[71]
Female Brown Norway rats (7 weeks)	30 mg/kg/day, orally, 10 months	↓lens opacities, blood glucose levels	[72]
30 diabetic dogs	2 mg/kg/day, orally 200 days	↑time to cataract formation; ↓lens opacities	[74]

Note: ↑, increase; ↓, decrease.

Overall, the multifaceted pharmacological properties of ALA, including its antioxidant activity, modulation of endothelial function, and inhibition of aldose reductase, highlight its potential as a promising adjunctive therapy in the comprehensive management of diabetes-related complications, such as neuropathy and retinopathy. Nevertheless, the majority of current evidence is derived from short-term clinical trials and animal model studies, with the long-term efficacy and safety of ALA remaining insufficiently established. Future studies should aim to elucidate the optimal dosing strategies, assess long-term safety profiles, and evaluate the clinical utility of molecular biomarkers, such as kallistatin, for personalized treatment monitoring, thereby minimizing adverse effects and enhancing therapeutic outcomes.

## 4. Novel Therapeutic Strategies of ALA

Despite its potential in treating various diseases, the clinical efficacy of ALA is limited due to challenges of low bioavailability and poor stability. Therefore, chemical modifications and advanced delivery systems have been explored to improve its stability and absorption.

### 4.1. Derivatives of ALA

**Figure 6:**
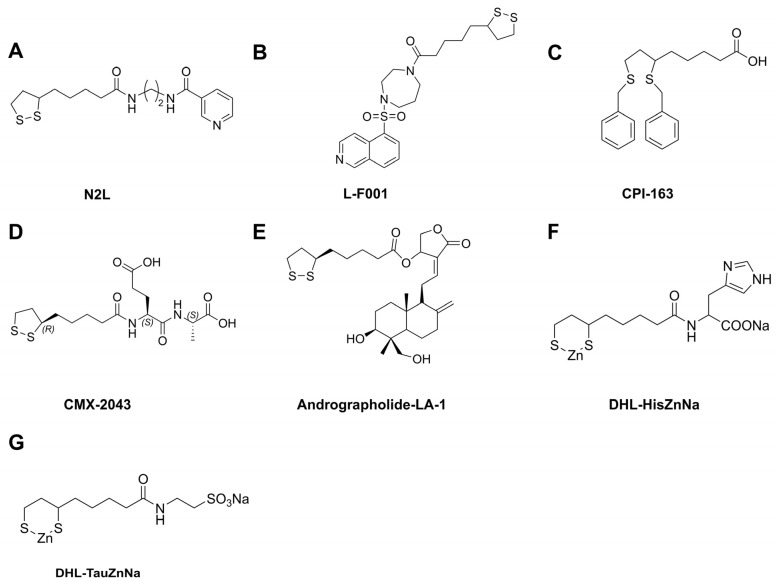
Structural characterization of ALA derivatives. (**A**) Chemical structure of N2L. (**B**) Chemical structure of L-F001. (**C**) Chemical structure of CPI-613. (**D**) Chemical structure of CMX-2043. (**E**) Chemical structure of andrographolide-LA-1. (**F**) Chemical structure of DHL-HisZnNa. (**G**) Chemical structure of DHL-TauZnNa.

Recent advances in pharmacological research have highlighted several ALA derivatives with promising therapeutic potential in various preclinical and clinical models due to their distinctive mechanisms (Figure 6).

#### 4.1.1. N2L

N2L (Figure 6A), a specific dimer of ALA formed by the linkage of two ALA molecules via a disulfide bond, was synthesized by Chen et al. [75] in 2014 with the aim of enhancing the biological activity of ALA and improving its therapeutic properties. N2L has been shown to exert neuroprotective effects against Aβ1–42-induced cytotoxicity by restoring the activities of GPX, SOD, and CAT to suppress oxidative stress, while inhibiting apoptosis by upregulating BCL-2 protein levels and downregulating cleaved Caspase-3 and BAX protein expression [76]. Additionally, high doses of N2L (>100 mol/L) decrease oxidative damage to hRPE cells exposed to blue light through the upregulation of BCL-2 and downregulation of Caspase-3 and BAX [77]. As a potent and selective high-affinity agonist of the niacin receptor GPR109A, N2L has demonstrated favorable effects in regulating lipid metabolism and inhibiting atherosclerosis. It also exhibited excellent antioxidant properties, maintaining a favorable safety profile [78]. Furthermore, N2L protects HT22 cells from RSL3-induced ferroptosis by inhibiting activation of the JNK/ERK signaling pathway, diminishing lipid peroxidation through the restoration of GPX4 expression, and the reduction of ACSL4 and COX-2 protein levels [79].

#### 4.1.2. L-F001

L-F001 (Figure 6B), a multifunctional fasudil-ALA dimer, effectively prevents paraquat-induced apoptosis by modulating GRP78 and CHOP expression, alleviating mitochondrial dysfunction, and reducing endoplasmic stress in PC12 cells [80]. It also protects against 6-OHDA-induced PC12 cell death and mitigates MPTP-induced dopaminergic neurotoxicity in mice by activating AKT and GSK-3β phosphorylation, as well as inducing the nuclear translocation of NRF2 and expression of HO-1 in a concentration- and time-dependent manner [81]. Additionally, L-F001 prevents RSL3-induced ferroptosis by inhibiting JNK activation and maintaining iron homeostasis, which reduces intracellular ROS and lipid peroxidation levels [82]. Furthermore, L-F001 protects against hypoxic–ischemic brain damage by reducing COX-2 and iNOS expression through inactivation of the TLR4 signaling pathway in HD rats [83].

#### 4.1.3. CPI-613

CPI-613 (Figure 6C), a multitargeted metabolic inhibitor derived from endogenous ALA, targets the dysregulated tricarboxylic acid (TCA) cycle in cancer cells by inhibiting both the α-ketoglutarate dehydrogenase (KGDH) and pyruvate dehydrogenase complex (PDC), which is currently undergoing clinical trials in various malignancies [84]. It has been found that CPI-613 prevents pancreatic cancer progression by stimulating ROS-mediated apoptosis through activation of the AMPK signaling pathway [85]. Additionally, the combination of CPI-613 with 5-fluorouracil or irinotecan disrupts the MMP and markedly impairs mitochondrial respiration, leading to colorectal cancer cell death in a Bim-dependent manner [86]. Another study has suggested that CPI-613 enhances the chemotherapy sensitivity of ovarian cancer cells by inducing mitochondrial dysfunction, triggering mitochondria-mediated apoptosis [87]. Furthermore, CPI-613 suppresses the proliferation of multiple myeloma cells by inducing mitochondrial metabolic dysfunction through the targeting of pyruvate dehydrogenase E1 subunit alpha 1 (PDHA1) and oxoglutarate dehydrogenase (OGDH), and it produces significant anti-tumor effects when combined with bortezomib at a lower dose [88].

#### 4.1.4. Others

Beyond the widely studied derivatives, other ALA derivatives have also been reported for the management of various diseases. CMX-2043 (Figure 6D), a novel ALA analog, appears to be a promising candidate for mitigating myocardial IR injury. Treatment with CMX-2043 was found to reduce myocardial IR injury regardless of whether it was administered prior to ischemic injury or during reperfusion. Notably, the most effective administration was observed when CMX-2043 was given 15 min prior to ischemia, indicating a specific therapeutic window where the compound may exert its protective effects most effectively [89]. Additionally, a phase II clinical trial demonstrated that CMX-2043 significantly reduced myocardial damage during percutaneous coronary intervention procedures in 142 patients who were randomized to receive either a single intravenous dose of CMX-2043 or a placebo 15–60 min before surgery [90]. Andrographolide-LA-1 (Figure 6E), a compound synthesized through the esterification of andrographolide and ALA, was proposed to be a major transcription factor that significantly reduces the inflammatory response in inflammatory bowel disease [91]. A phase II clinical trial has demonstrated that although the application of 1% DHL-HisZnNa (Figure 6F) does not inhibit chemotherapy-induced alopecia (CIA) in breast cancer patients, it promotes the recovery from CIA [92]. Moreover, DHL-HisZnNa treatment inhibits colorectal cancer cell proliferation through increased p53 phosphorylation and p21 protein levels, as well as decreased levels of phosphorylated retinoblastoma (Rb) [93]. The continuous subcutaneous administration of DHL-TauZnNa (Figure 6G) for 41 days significantly inhibited colon cancer cell proliferation via G2/M cell cycle arrest and the induction of excessive autophagy [94].

The development of ALA derivatives has expanded the therapeutic potential of ALA beyond its antioxidant properties. These derivatives exhibit a diverse range of mechanisms, including antioxidant activity, ferroptosis inhibition, mitochondrial dysfunction, and the modulation of key signaling pathways, such as AKT, GSK-3β, NRF2, and TLR4. These data suggest that ALA derivatives hold promise for treating a variety of diseases, from neurodegenerative disorders to cardiovascular diseases and cancers. Further clinical trials and mechanistic studies are needed to fully elucidate the therapeutic efficacy and safety profiles of these novel compounds.

### 4.2. Delivery Systems of ALA

The amphiphilicity of ALA facilitates efficient cellular uptake and membrane penetration, enhancing its effectiveness as both an antioxidant and a component in drug formulations. Furthermore, ALA’s ability to form disulfide-linked dimers plays a crucial role in stabilizing drug delivery systems. These unique properties position ALA as a promising candidate for optimizing drug delivery systems.

#### 4.2.1. Nanoparticles

**Figure 7:**
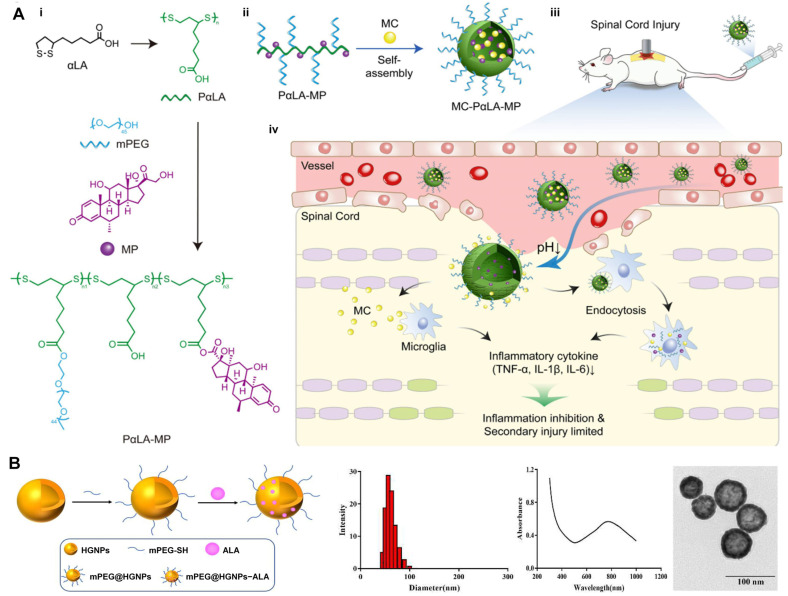
Delivery of ALA via nanoparticles. (**A**) The construction and function of MC-PαLA-MP NPs in TSCI rats, including the synthesis of PαLA-MP (**i**), self-assembly of MC-PαLA-MP NPs (**ii**) and its anti-inflammatory mechanism in TSCI rats (**iii**,**iv**) [98]. Copyright: Int. J. Nanomed. 2022. (**B**) The preparation of mPEG@HGNPs-ALA and characterization of its size, absorption spectrum, and morphology. Reproduced with permission [99]. Copyright: Artif. Cells Nanomed. Biotechnol. 2023. MC, minocycline; PαLA, poly (α-lipoic acid); MP, methylprednisolone; NPs, nanoparticles; TSCI, traumatic spinal cord injury; mPEG, methoxy poly(ethylene glycol); HGNPs, hollow gold nanoparticles.

Nanoparticle (NP)-based systems have been shown to enhance the stability and bioavailability of ALA [95]. Liposomes encapsulating R-ALA (LIP/RLA) have demonstrated enhanced oral absorption and bioavailability, improving hepatoprotective effects in rats with liver injury [96]. The administration of AlCl3 significantly elevates neuroinflammation, oxidative stress, and apoptosis, while reducing brain fatty acid content. These adverse effects were effectively ameliorated by ALA, ALA-loaded chitosan nanoparticles (ALA-CsNPs), and ALA-loaded solid lipid nanoparticles (ALA-SLNPs). Notably, the ALA nanoparticles exhibit more pronounced therapeutic effects compared to free ALA [97]. Additionally, the systemic administration of MC-PαLA-MP NPs for 8 weeks provides effective neuroprotection and preserves motor function by alleviating inflammation after experimental traumatic spinal cord injury in rats (Figure 7A) [98]. PEGylated hollow gold nanoparticles loaded with ALA (mPEG@HGNPs-ALA) effectively scavenge ROS induced by H_2_O_2_ injury and enhance cell viability, thereby facilitating the treatment of osteoporosis (Figure 7B) [99].

#### 4.2.2. Hydrogels

**Figure 8:**
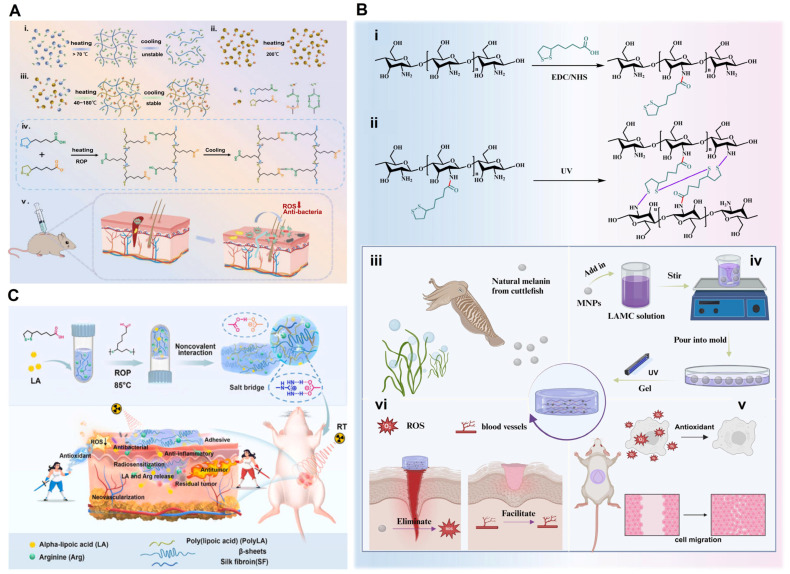
Delivery of ALA via a hydrogel. (**A**) The construction, structural characteristics, and functions of LA-DESP. The preparation and structure of polyLA (**i**), thermal stability of LA-Na (**ii**), preparation, stabilization mechanism, and chemical structure of LA-DESP (**iii**,**iv**), and its function promoting skin wound healing (**v**). Reproduced with permission [100]. Copyright: Adv. Funct. Mater. 2023. (**B**) The construction, chemical structural, and functions of LAMC@MNPs. Synthesis of LAMC (**i**), formation process of LAMC hydrogel (**ii**), sources of natural melanin (**iii**), construction of LAMC@MNPs hydrogel (**iv**), and its role in diabetic wounds (**v**,**vi**). Reproduced with permission [102]. Copyright: Carbohydr. Polym. 2024. (**C**) Preparation of PolyLA-based hydrogel and its application in breast cancer recurrence and skin injuries after radiotherapy [105]. Copyright: Bioact. Mater. 2024. DESP, deep eutectic supramolecular polymer; LAMC, α-lipoic acid-modified chitosan; MNPs, melanin nanoparticles.

Hydrogel systems that encapsulate ALA or its derivatives enable controlled release and offer enhanced therapeutic benefits in various diseases, owing to their potent biological activity. A self-stabilized deep eutectic supramolecular polymer (LA-DESP) prepared in one step by heating a mixture of AL and ALA-Na demonstrated rapid and strong adhesion to various substrates. It functions as a tissue adhesive, replacing surgical sutures and promoting wound healing (Figure 8A) [100]. Furthermore, an injectable hydrogel based on hyaluronic acid, chitosan, and potassium-γ-cyclodextrin metal-organic frameworks (K-γ-CD-MOFs) loaded with ALA demonstrated both antioxidant and antibacterial properties. By promoting cell migration and proliferation, it effectively mitigated oxidative stress-induced cellular damage, facilitating the healing of chronic full-thickness skin wounds [101]. ALA-modified chitosan (LAMC) and melanin nanoparticle (MNP) (LAMC@MNPs) hydrogels not only exhibit excellent skin adhesion but also effectually attenuate oxidative stress by scavenging radicals, which accelerates wound healing (Figure 8B) [102]. Additionally, ALA and trimethylglycine expeditiously form supramolecular hydrogels at room temperature, exhibiting injectability and the potential for 3D printing. A hydrogel bandage derived from this system significantly enhanced wound healing by facilitating wound closure, creating a protective physical barrier and providing anti-inflammatory effects [103]. Furthermore, poly (lipoic acid-co-sodium lipoic acid) (PLAS) was directly obtained in aqueous solution via ring-opening polymerization triggered by heat and concentration, benefiting from the dynamic disulfide bonds in ALA. PLAS hydrogels exhibited promising antioxidant efficiency, efficiently scavenging intracellular ROS and protecting against spinal cord injury [104]. A multifunctional poly (lactic acid)-based hydrogel was constructed through the one-step heating of an ALA/arginine/silk fibroin mixture. This hydrogel, when implanted into the body or applied to the skin of mice, effectively prevented postoperative tumor recurrence and facilitated the treatment of radiation-induced skin injuries after radiotherapy in breast cancer (Figure 8C) [105]. When incorporating COS into an ALA hydrogel (COS@LA-hydrogel), as depicted, this hydrogel was gradually degraded in the reducing environment at the implantation site, releasing both LA and COS. The combined action of LA and COS on tumor cells inhibits the phosphorylation of AKT within the PI3K–AKT pathway, thereby preventing the progression of residual tumor cells and resulting in a pronounced antitumor effect [106].

#### 4.2.3. Electrospinning

**Figure 9:**
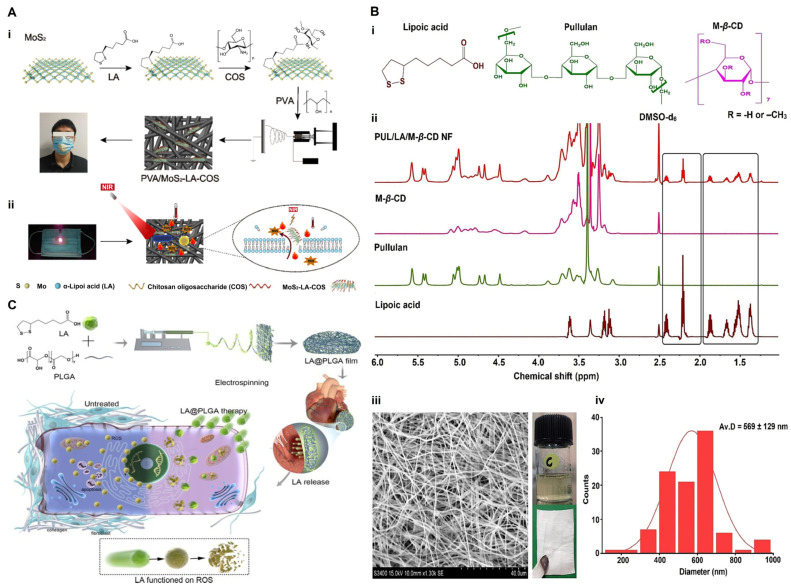
Delivery of ALA via electrospinning. (**A**) The construction and function of the PVA/MoS2-LA-COS nanofiber membrane. The preparation and antibacterial function of PVA/MoS2-LA-COS as a photothermal agent (**i**), and photothermal inactivation mechanism under NIR irradiation (**ii**). Reproduced with permission [109]. Copyright: J. Nanostructure Chem. 2024. (**B**) Preparation and characterization of PUL/LA/M-β-CD NF. Preparation process of PUL/LA/M-β-CD NF with LA, PUL, and M-β-CD (**i**); the highlighted portion represents the characteristic peaks of ALA observed in ALA and PUL/LA/M-β-CD NF (**ii**), morphology under scanning electron microscopy (**iii**), and average fiber size distribution (**iv**). Reproduced with permission [110]. Copyright: Int. J. Biol. Macromol. 2024. (**C**) Preparation of LA@PLGA copolymers for the blockade of ROS production in cardiomyocytes, reducing apoptosis and fibrosis, thereby improving cardiac function in mice with AMI [111]. Copyright: Int. J. Pharm. 2023. PVA, poly(vinyl alcohol); COS, chitosan oligosaccharide; PUL, pullulan; M-β-CD, methyl-β-cyclodextrin (M-β-CD); PLGA, poly(lactic-co-glycolic); ROS, reactive oxygen species; AMI, acute myocardial infarction.

Electrospinning is a widely recognized technique for fabricating micrometer-thick films consisting of submicron nanofibers and polymers, which allows for precise manipulation of the fiber diameter and distribution, making it highly suitable for biomedical applications [107]. Recently, an electrospinning technique has been employed to prepare nanofiber-based delivery systems, further enhancing the efficacy of ALA in treating oxidative stress and inflammatory conditions [108]. A nano-flower-like MoS2, synthesized via electrochemical methods, was functionalized with ALA and chitosan oligosaccharide to create a composite material (MoS2-LA-COS) with high biocompatibility, good dispersion, and near-infrared (NIR) light responsivity. This material was effectually attached to an electrospun nanofiber membrane, endowing the fiber scaffold with excellent photothermal performance and exhibiting significant antibacterial effects under NIR light irradiation (Figure 9A) [109]. The electrospun nanofibers (PUL/LA/M-β-CD NF), prepared by combining pullulan (PUL) with LA-M-β-CD, effectively reduced the production of ROS by downregulating COX-2 and iNOS in LPS-treated RAW 264.7 cells. Additionally, these nanofibers inhibited pro-inflammatory cytokine expression (IL-1β, TNF-α, and IL-6) and NF-κB nuclear translocation, highlighting the significant potential for oral anti-inflammatory treatment (Figure 9B) [110]. Poly (lactic acid-co-glycolic acid) (PLGA) copolymers were utilized as carriers to form films (LA@PLGA) via electrospinning, enabling the controlled release of ALA and effectively blocking the production of ROS in damaged hearts. Thus, LA@PLGA significantly improved cardiac function in mice with acute myocardial infarction through its strong anti-apoptotic and antioxidant activity (Figure 9C) [111].

The integration of ALA into various advanced drug delivery systems, such as nanoparticles, hydrogels, and electrospun nanofibers, has enhanced its therapeutic efficacy in treating a wide range of diseases, including neurodegenerative disorders, cancer, cardiovascular diseases, and chronic wounds. These delivery systems leverage ALA’s unique properties, such as its amphiphilicity, antioxidant capacity, and ability to form disulfide-linked dimers, to optimize drug stability, bioavailability, and controlled release. Future research should focus on optimizing the design and fabrication of these systems to improve their therapeutic outcomes, explore their potential in clinical settings, and further investigate their safety profiles.

## 5. Conclusions

The unique ability of ALA to function as both a hydrophilic and hydrophobic compound enhances its versatility as a therapeutic agent. This dual characteristic enables ALA to target a wide range of cellular and molecular processes, positioning it as an available therapeutic option for treating complex diseases with multifactorial pathologies. However, the relatively low bioavailability remains a challenge for the therapeutic application of ALA. The development of novel ALA derivatives and the incorporation of ALA into biomaterials provide novel and promising strategies to improve the efficacy and therapeutic potential of ALA-based therapies. Breakthroughs in these technologies not only overcome the inherent limitations of ALA but also expand its potential applications in modern medicine.

Despite the considerable therapeutic potential of ALA in diverse diseases, further investigation is required to address the challenges and opportunities it presents. First, the clinical efficacy can be improved by optimizing the bioavailability and pharmacokinetics of ALA through innovative therapies, such as advanced ALA derivatives or delivery systems. Second, additional mechanistic studies are necessary to elucidate the precise molecular pathways by which ALA and its derivatives, particularly in multifactorial diseases like cardiovascular diseases and cancer. Additionally, large-scale, well-designed clinical trials are essential to validate the preclinical findings and establish standardized dosing regimens. Exploring synergistic combinations of ALA with existing therapeutic agents may also offer new treatment strategies. Finally, a comprehensive evaluation of the long-term safety and potential adverse effects of ALA-based interventions are critical for their successful translation into clinical practice. Addressing these gaps will not only advance our understanding of ALA but also broaden its potential applications in precision medicine.

## Figures and Tables

**Table 1:** Therapeutic mechanisms of ALA in neurodegenerative diseases.

Cells Line/Animal	ALA Treatment	Mechanism	Reference
Alzheimer’s disease			
SH-SY5Y cells	100 µM, 1 mM, 24 h	↑ATP, MMP; ↓ROS	[24]
SH-SY5Y cells; Fruit fly	0–50 µM, 24 h; 2 mM, ALA-food, 14 days	↑Copper metabolism, Locomotor activity; ↓Eye photoreceptor cells	[25]
Male Wistar rats (160–180 g)	30 mg/kg/day, injected intraperitoneally, 11 days	↑STL, DA, NA; ↓AChE activity	[26]
BV2 cells	100 µM, 24 h	↑BCL-2, IκB-α, SOD, GPX, CAT, p-GSK3β, β-catenin; ↓BAX, Caspase-3, NF-κB, p65, GSK3β, p-β-catenin	[27]
PC12 cells	1, 10, 100 µM, 48 h	↑BCL-2, IκB-α, Frizzled 2, p-GSK3β, β-catenin; ↓BAX, Caspase-3, NF-κB, p65, GSK3β, p-β-catenin	[28]
20E2 cells; APP23/PS45 transgenic mice (2 months)	400 µM, 24 h; 5 mg/kg/day, injected intraperitoneally, 4 months	↑ALAM10, C83, LC3, BNIP3L; ↓APP, C89, C99, P62	[29]
Parkinson’s disease			
PC12 cells; Mice	10 µM, 24 h; 50 mg/kg/day, injected intraperitoneally, 14 days	↑FTH1, GPX4, x-CT, SIRT1, NRF2; ↓DMT1, ROS	[30]
Male Sprague–Dawley (SD) rats (250–300 g)	100 mg/kg/day, injected intraperitoneally, 14 days	↑SOD, GSH; ↓ROS, Iron, TH, IRP2, DMT1	[31]
Male Sprague–Dawley (SD) rats (250–300 g)	30 mg/kg/day, injected intraperitoneally, 7 days	↑GSH, PGC1α, TFAM, NRF2; ↓MDA, IL-6	[32]
SH-SY5Y cells; Male C57BL/6 mice (22–27 g)	200 µM, 24 h; 50 mg/kg/day, injected intraperitoneally, 14 days	↑SIRT1, PGC1α, TH, SOD; ↓MDA, DCF	[33]
Male C57BL/6 mice (23–28 g)	50 mg/kg/day, injected intraperitoneally, 14 days	↓NF-κB, TNF-α, iNOS	[34]
PC12 cells	0, 0.1, 1, 10 and 20 mM, 24 h	↑SLC7A11, GPX4, p-P13k, p-AKT, NRF2	[35]
43 AD patients	600 mg/kg/day, orally, 48 months	↑MMSE, ADAScog	[36]
39 AD patients	600 mg/kg/day, orally, 12 months	↑MMSE, IADL	[37]

Note: ↑, increase; ↓, decrease.

**Table 2:** Therapeutic mechanisms of ALA in pulmonary diseases.

Cells Line/Animal	ALA Treatment	Mechanism	Reference
Pulmonary fibrosis			
Male C57BL/6 J mice (8 weeks)	3.0 mg/mL, gavage, 28 days	↑TFAM, COX-4, ATPase, p-AMPKα, PGC1α, NRF2; ↓TGF-β1, α-SMA, PAI-1, COL1-α1, COL3-α1, KEAP-1	[40]
C57BL/6J mice	100 mg/kg/day, orally, 28 days	↑E-cadherin, TAC, CAT, GSH, ATP, PPARα, TFAM; ↓Collagen, α-SMA, Vimentin, MDA, ROS	[41]
Male Wistar rats (125–130 g)	100 mg/kg/day, orally, 21 days	↑GSH, TAC; ↓TGF-β1, IFN-γ, ALT, AST, MTC, α-SMA	[42]
C57BLKS/J mice (8 weeks)	Overexpressed LIAS gene	↑NRF2, P65, IKK-β, I-κB; ↓TNF-α, TGF-β1, Vimentin, α-SMA, PAI-1, COL1-α1, COL3-α1	[43]
Male albino rats (200–240 g)	200 mg/kg/day, injected intraperitoneally, 6 weeks	↑COX-2, SOD, GPX; ↓NOX4, TNF-α, IL-6, IL-1β, IL-10, α-SMA, Caspase 3, NOX-4	[44]
Mice	200 mg/kg/day, gavage, 2 weeks	↓Inflammation, Pulmonary edema, Collagen deposition.	[45]
Non-small cell lung cancer			
A549 cells; Nude mice (6 weeks)	5 mM, 24 h; 50 mg/kg/day, orally, 18 days	↑LDH, BAX/BCL-2, p62, p-p70S6K/p70S6K, p-mTOR/mTOR; ↓LC3-II, VPS34, Beclin-1, ATG13	[46]
H460, H292, H23 cells	0–5 μM, 48 h	↓CD133, ALDH1A1, CD44, Oct-4, β-catenin, p-Akt, EMT (E-cadherin, Vimentin, Snail, Slug)	[47]
NCI-H1975, A549 cells	2.0 mM, 24 h	↓Grb2, p-EGFR, p-ERK, CDK2/4/6, Cyclin D3/E1	[48]
A549, PC9 cells	1.5 mM, 24 h or 48 h	↑ROS, Caspase-9; ↓BCL-2, PDK1, NRF2	[49]
H460 cells	0–10 μM, 48 h	↓Integrin β1 and β3, p-AKT, BCL-2	[50]

Note: ↑, increase; ↓, decrease.

**Table 3:** Therapeutic mechanisms of ALA in cardiovascular diseases.

Cells Line/Animal	ALA Treatment	Mechanism	Reference
Myocardial infarction			
C57Bl/6 mice	75 mg/kg/day, orally, 7 days	↑LVEF; ↓LVR, MDA, NOX, MPO	[52]
H9C2, RAW264.7 cells; Male C57BL/6J mice (20–25 g)	100 μM,12 h; 30 mg/kg/day, injected intraperitoneally, 7 days	↑IL-10, TGF-β, BCL-2, p62, CD206; ↓IL-1β, IL-6, HMGB1, NF-κB. BAX, LC3 II/LC3 I	[53]
Male Wistar rats (400–450 g)	100 mg/kg/day, gavage, 14 days	↓TNF-α, IL-1β, IL-6, Caspase-3, BAX, Cyt-c	[54]
Wistar rats of both sexes (150–170 g)	50 mg/kg/day gavage, 10 days	↑SOD, CAT; ↓Incidence of mortality, CK-MB, MDA	[55]
Myocardial ischemia–reperfusion			
Male Wistar rats (22–24 months)	10 mg/kg/day, gavage, 14 days	↑MFN1, MFN2, FOXO1; ↓LDH, IS, DRP1, FIS1	[56]
Male Wistar rats (200–250 g)	100 mg/kg/day, orally, 5 weeks	↑NO, Connexin-43; ↓PFC, VF	[57]
Male Wistar rats (200–250 g)	100 mg/kg/day, orally, 5 weeks	↓Mitochondrial ROS, LC3, p62	[58]
Male Wistar rats (200–250 g)	100 mg/kg/day, orally, 5 weeks	↑BCL-2; ↓BAX, cleaved Caspase-3	[59]
H9C2 cells; Male Sprague–Dawley (SD) rats (250 g ± 10 g)	100 Μm, 12 h; 30 mg/kg/day, injected intraperitoneally	↑SOD, BCL-2; ↓IL-6, TNF-α, IL-1β, BAX, HMGB1, TLR4, NF-κB	[60]
Heart failure			
Sprague–Dawley rats	50 mg/kg/day, injected intraperitoneally, 2 weeks/month for 9 months	↑GSH/GSSG, TAC, NOX; ↓MDA, TNF-α, IL-6	[61]
NRCMs cells; Male C57BL/6 mice (20–25 g)	10 μM,48 h; 0.2% (wt/wt), drinking, 4 weeks	↑BCL-2, TOM20, ALDH2, FUNDC1, LC3, NRF1; ↓cleaved Caspase-3, BAX, P62	[62]

Note: ↑, increase; ↓, decrease.

## Data Availability

No new data were created or analyzed in this study. Data sharing is not applicable to this article.

## Abbreviations

The following abbreviations are used in this manuscript:

AChE	Acetylcholinesterase
ABI	Ankle Brachial Index
ADMA	Asymmetric dimethylarginine
ADAScog	Alzheimer’s disease assessment scale—cognitive subscale
ALAM10	Amyloid precursor protein-like protein 10
ALDH1A1	Aldehyde dehydrogenase 1 family member A1
ALDH2	Aldehyde dehydrogenase 2
ALT	Alanine aminotransferase
AMPK	AMP-activated protein kinase
AMI	Acute myocardial infarction
APP	Amyloid precursor protein
ATP	Adenosine triphosphate
ATG13	Autophagy-related 13
ACSL4	Acyl-coA synthetase long chain family member 4
AR	Aldose reductase
AGEs	Advanced glycation end products
BAX	BCL-2-associated x protein
BCL-2	B-cell lymphoma 2
BNIP3L	BCL2/adenovirus E1B 19 kDa interacting protein 3-like
Caspase-3	Cysteine-aspartic protease 3
CAS	Composite autonomic score
CAT	Catalase
CHO	C/EBP homologous protein
CD133	Cluster of differentiation 133
CD44	Cluster of differentiation 44
CK-MB	Creatine kinase-mb
COX	Cyclooxygenase
COL1-α1	Collagen type I alpha 1 chain
COL3-α1	Collagen type III alpha 1 chain
COS	Chitosan oligosaccharide
CPT	Current perception threshold
Cyt-c	Cytochrome c
DESP	Deep eutectic supramolecular polymer
DN4	Douleur neuropathique en 4 questions
DRP1	Dynamin-related protein 1
DPN	Diabetic peripheral neuropathy
DSPN	Diabetic sensorimotor polyneuropathy
ERK	Extracellular signal-regulated kinase
ERI	Erythropoietin resistance index
FIS1	Mitochondrial fission 1 protein
FBG	Fasting blood-glucose
FMD	Flow-mediated dilation
FOXO1	Forkhead box O1
FRAP	Ferric reducing antioxidant power
Frizzled	Frizzled class receptor 2
FUNDC1	Fun14 domain containing 1
GPR109A	G protein-coupled receptor 109A
FTH1	Ferritin heavy chain 1
GSK3β	Glycogen synthase kinase 3 beta
GSH	Glutathione
GPX	Glutathione peroxidase
GRB2	Growth factor receptor bound protein 2
HMGB1	High mobility group box 1
HbA1	Glycosylated hemoglobin
HGNPs	Hollow gold nanoparticles
IADL	Instrumental activities of daily living
LAMC	Alpha-lipoic acid-modified chitosan
IL-1β	Interleukin 1 beta
IL-6	Interleukin 6
IL-10	Interleukin 10
IS	Infarct size
IKK-β	Inhibitor of κB kinase beta
IRP2	Iron regulatory protein 2
IκB-α	Inhibitor of kappa B alpha
JNK	c-Jun N-terminal kinase
LC3	Microtubule-associated protein 1 light chain 3
LDH	Lactate dehydrogenase
LVEF	Left ventricular ejection fraction
MC	Minocycline
MDA	Malondialdehyde
MFN	Mitofusin
MMP	Matrix metalloproteinase
MMSE	Mini-Mental State Examination
MNPs	Melanin nanoparticles
mPEG	methoxy poly(ethylene glycol)
MPO	Myeloperoxidase
M-β-CD	Methyl-β-cyclodextrin (M-β-CD)
NADPH	Nicotinamide adenine dinucleotide phosphate
NIS-LL	Neuropathy impairment score of the lower limbs
NSC	Neuropathy symptoms and change
NPs	Nanoparticles
NO	Nitric oxide radical
NOX-4	NADPH oxidase 4
NDS	Neurological deficit score
NSS	Neurological symptom score
NTSS-6	Neurological total symptom score-6
NF-κB	Nuclear factor kappa B
NRF2	Nuclear factor erythroid 2-related factor 2
p70S6K	Ribosomal protein S6 kinase (S6K1)
PAI-1	Plasminogen activator inhibitor-1
PDK1	Pyruvate dehydrogenase kinase 1
PGC1α	Peroxisome proliferator-activated receptor gamma coactivator-1alpha
PPARα	Peroxisome proliferator-activated receptor alpha
p-AKT	Phosphorylated protein kinase B
p-PI3k	Phosphorylated phosphoinositide 3-kinase
PFC	Pulmonary function capacity
ROS	Reactive oxygen species
6-OHDA	6-Hydroxydopamine
SIRT1	Sirtuin 1
SLC7A11	Solute carrier family 7 member 11
SOD	Superoxide dismutase
SMAD3	SMAD family member 3
TAC	Total antioxidant capacity
TGF-β1	Transforming growth factor beta 1
TLR4	Toll-like receptor 4
TNF-α	Tumor necrosis factor alpha
TOM20	Translocase of outer mitochondrial membrane 20
TSCI	Traumatic spinal cord injury
VAS	Visual analog scale
VPT	Vibration perception threshold
8-OHdG	8-hydroxy-2′-deoxyguanosine
